# Brain microvascular endothelial cell dysfunction in an isogenic juvenile iPSC model of Huntington’s disease

**DOI:** 10.1186/s12987-022-00347-7

**Published:** 2022-06-30

**Authors:** Raleigh M. Linville, Renée F. Nerenberg, Gabrielle Grifno, Diego Arevalo, Zhaobin Guo, Peter C. Searson

**Affiliations:** 1 Institute for Nanobiotechnology, Johns Hopkins University, Baltimore, MD USA; 2 Department of Biomedical Engineering, Johns Hopkins University, Baltimore, MD USA; 3 Department of Materials Science and Engineering, Johns Hopkins University, Baltimore, MD USA

**Keywords:** Blood–brain barrier, Huntington’s disease, Brain microvascular endothelial cells, Neurodegenerative disease, Induced pluripotent stem cells

## Abstract

Huntington’s disease (HD) is an inherited neurodegenerative disease caused by expansion of cytosine–adenine–guanine (CAG) repeats in the huntingtin gene, which leads to neuronal loss and decline in cognitive and motor function. Increasing evidence suggests that blood–brain barrier (BBB) dysfunction may contribute to progression of the disease. Studies in animal models*, *in vitro models, and post-mortem tissue find that disease progression is associated with increased microvascular density, altered cerebral blood flow, and loss of paracellular and transcellular barrier function. Here, we report on changes in BBB phenotype due to expansion of CAG repeats using an isogenic pair of induced pluripotent stem cells (iPSCs) differentiated into brain microvascular endothelial-like cells (iBMECs). We show that CAG expansion associated with juvenile HD alters the trajectory of iBMEC differentiation, producing cells with ~ two-fold lower percentage of adherent endothelial cells. CAG expansion is associated with diminished transendothelial electrical resistance and reduced tight junction protein expression, but no significant changes in paracellular permeability. While mutant huntingtin protein (mHTT) aggregates were not observed in HD iBMECs, widespread transcriptional dysregulation was observed in iBMECs compared to iPSCs. In addition, CAG expansion in iBMECs results in distinct responses to pathological and therapeutic perturbations including angiogenic factors, oxidative stress, and osmotic stress. In a tissue-engineered BBB model, iBMECs show subtle changes in phenotype, including differences in cell turnover and immune cell adhesion. Our results further support that CAG expansion in BMECs contributes to BBB dysfunction during HD.

**Supplementary Information:**

The online version contains supplementary material available at 10.1186/s12987-022-00347-7.

## Introduction

Huntington’s disease (HD) is an inherited autosomal dominant neurodegenerative disease that affects 1 in 10,000 Americans and causes cognitive deficits and loss of motor function that are ultimately fatal [1]. HD is caused by the expansion of cytosine–adenine–guanine (CAG) repeats in the huntingtin gene (*HTT*), which leads to the production of mutant huntingtin protein (mHTT). Both transcriptional and protein-level dysfunction from this mutation contribute to neuronal loss, accompanied by cognitive and motor dysfunction [2].

The human blood–brain barrier (BBB) is comprised of brain microvascular endothelial cells (BMECs), along with supporting cells, that maintain neuronal homeostasis. There is accumulating evidence across in vitro and in vivo studies that HD is associated with dysfunction of the BBB [3–6]. Cerebrovascular changes are observed in animal models and post-mortem human tissue including increased microvascular density [3, 7, 8], BBB breakdown [3, 4], and altered cerebral hemodynamics [8–10]. Additionally, in vitro studies using human induced pluripotent stem cell (iPSC)-derived BMEC-like cells (iBMECs) have found that CAG expansion elevates angiogenic potential, reduces paracellular barrier strength, and changes transcellular transport [5, 6]. These cerebrovascular changes may contribute to the early pathogenesis of HD and represent a possible therapeutic target. However, many aspects of BMEC phenotype remain to be explored and, to date, isogenic controls have not been utilized for these studies. To expand the understanding of changes in BMEC phenotype that may contribute to HD pathogenesis, we build on previous reports by: (1) utilizing an isogenic pair of iPSCs to directly determine effect of CAG mutation on differentiation trajectory and resulting iBMEC phenotype, (2) utilizing two-dimensional (2D) and three-dimensional (3D) in vitro models to confirm results in the presence of physiological cues (i.e. shear stress) and to broaden the repertoire of functional measurements, and (3) by validating our results across multiple differentiations and protocol variables.

We differentiated iBMECs from a juvenile HD patient with 180 CAG repeats and an isogenic control in which the CAG expansion was corrected using CRISPR/Cas9 gene editing [6]. Recent work suggests a neurodevelopmental component to HD progression [11], while juvenile-derived CRISPR/Cas9-corrected iPSCs were previously used to show reversal of phenotypic abnormalities in iPSC-derived neurons [12]. Our approach is distinct from existing work utilizing adult HD iPSCs [5], which harbor aged-induced epigenetic changes [13, 14]. We found that CAG expansion reduced transendothelial electrical resistance (TEER) of HD iBMECs (~ three-fold), corresponding with reduced localization of tight junction proteins, but no difference in paracellular permeability to small and large molecular weight compounds. Furthermore, we confirmed that CAG expansion was associated with reduced TEER across differentiation variables (seeding density, Transwell seeding density, and media composition). Critically, other aspects of BMEC phenotype were altered by CAG expansion including decreased efflux activity, increased sensitivity to angiogenic, oxidative, and osmotic factors, dysregulated cell turnover, and increased immune cell adhesion.

## Materials and methods

### Cell culture

Four induced pluripotent stem cell (iPSC) sources were used in this work: juvenile-onset HD iPSCs with 180 CAG repeats (HD180) [12], isogenic CRISPR-corrected controls of HD180 with 18 CAG repeats (HD-corrected) [12], non-isogenic adult onset HD iPSCs with 50 CAG repeats (HD50) (from NINDS cell repository #NN0003930), and non-isogenic control iPSCs with 21 CAG repeats (HD21) (from Allen Cell Institute #AICS-0023). The two isogenic cells were provided by the Pouladi Lab at the National University of Singapore. Details of each cell line are summarized in Additional file 2: Table S1. Cell culture was performed at 37 °C and 5% CO_2_. iBMECs were differentiated from iPSCs using protocols developed in the Searson Lab [15, 16]. Briefly, iPSC colonies were formed on six-well plates by seeding iPSCs singularized with Accutase (Invitrogen #A1110501) at 10,000 cells cm^−2^ (additional seeding densities outlined below) and growing for 3 days in either mTeSR™1 or TeSR™-E8™ (Stem Cell Technologies #85850 and #05990). Note that these media were not used interchangeably. Culture plates were coated with 83 µg mL^−1^ growth factor reduced basement membrane matrix (Matrigel; Corning #354230) in DMEM/F12 (ThermoFisher 11320033) for 1 h at room temperature. Colonies were then treated with UM/F- media: DMEM/F12, 20% KnockOut™ serum replacement (ThermoFisher #10828028), 1% non-essential amino acids (ThermoFisher #11140050), 0.5% GlutaMAX™ (ThermoFisher #35050061), and 0.836 μM beta-mercaptoethanol (ThermoFisher #21985023) for 6 days, and then in endothelial media: human endothelial cell serum-free medium (ThermoFisher #11111044), 1% human serum from platelet poor human plasma (Sigma-Aldrich #P2918), 2 ng mL^−1^ bFGF (Fisher Scientific #233FB025CF), and 10 μM all-trans retinoic acid (Sigma-Aldrich #R2625) for 2 days. iPSC medium was switched daily using a volume of 2 mL; UM/F- and endothelial media were switched daily using a media volume of 1 mL. At various stages of the differentiation, viable cells were manually counted on a hemacytometer based on Trypan blue (Corning #25-900-Cl) exclusion. At the end of the differentiation, cells were singularized using a 30-min treatment with Accutase. Adherent cells were isolated by sub-culture on a plate coated overnight with 50 μg mL^−1^ human placental collagen IV (Sigma #C5533) and 25 μg mL^−1^ fibronectin from human plasma (Sigma #F2006). This process was conducted for 1 h in endothelial media supplemented with 1% penicillin–streptomycin (ThermoFisher #15140122) and 10 μM ROCK inhibitor Y27632 (ATCC #ACS-3030). Following sub-culture, the monolayer of adherent cells was washed with phosphate-buffered saline (PBS; ThermoFisher #10010-023) and then singularized using a 10-min treatment with Accutase. Cells were then seeded onto collagen IV and fibronectin-coated surfaces at 0.33 × 10^6^ cells cm^−2^. For the first 24 h of culture, the media matched that of sub-culture but was then replaced with basal media (human endothelial cell serum-free medium, 1% human platelet poor plasma-derived serum, and 1% penicillin–streptomycin). HD180 and HD-corrected iPSCs were confirmed to be isogenic using the PowerPlex® 18D system (Promega). A luminescence-based MycoAlert™ Mycoplasma Detection Kit (Lonza #LT07-418) was used to confirm absence of mycoplasma.

### Differentiation variables

Beyond the differentiation scheme presented above, differentiation variables were adjusted to determine effects on outcomes and iBMEC phenotype. These variables included: (1) initial iPSC seeding density, (2) Transwell seeding density, (3) removal of the sub-culture step before seeding for experiments, (4) media volume used during differentiation, and (5) use of a serum-free medium alternative during differentiation and Transwell culture. To test the effect of initial seeding density on differentiation outcomes, hiPSCs were passed using the technique described previously, but seeded at densities of 5, 10, 20, 30, 40 × 10^3^ cells cm^−2^ in parallel on Matrigel-coated plates. To test the effect of Transwell seeding density on the barrier function of iBMECs, the cells were harvested using the technique described previously, and seeded on Transwells at the densities of 0.33 and 1 × 10^6^ cells cm^−2^ (three-fold difference in density) without the use of a sub-culture purification step. To determine the effect of the media volume used during differentiation, cells were grown in either 1 or 2 mL of UM/F- and RA media throughout the duration of the differentiation. To determine the effect of performing a serum-free differentiation, the 1% human platelet poor plasma-derived serum in endothelial media used in the final 2 days on the differentiation and during Transwell culture was replaced with 1 × B-27 Supplement (ThermoFisher #17504044), as previously demonstrated [17].

### Immunofluorescence

iBMECs were seeded at 250,000 cells cm^−2^ on borosilicate cover glass slides (coated with fibronectin and collagen IV as described above) and cultured for 2 days using media outlined above. iBMECs were then washed with 1 × PBS, fixed with ice cold methanol for 15 min, and blocked with 10% goat serum (Cell Signaling Technology #5425) or 10% donkey serum (Millipore Sigma #D9663) supplemented with 0.3% Triton X-100 (Millipore Sigma #108643) in PBS for 30 min. Primary antibodies are summarized in Additional file 2: Table S2. Cells were treated with Alexa Flour-647 and Alexa Flour-488 conjugated secondary antibodies (Life Technologies) diluted 1:200 in blocking buffer for 45 min at room temperature. To localize nuclei, cells were treated with 1 μg mL^−1^ DAPI (ThermoFisher #D1306). Between each step of the staining protocol, monolayers were washed three times with 1XPBS for 5 min. Images were acquired using a 40× magnification objective (Nikon) on an inverted microscope (Nikon Eclipse Ti-E) with illumination provided by an MLC400 monolithic laser combiner (Keysight Technologies). To enable semi-quantitative analysis of protein levels, we normalized fluorescence signal to nuclear signal across at least four biological replicates for each cell source.

### RNA sequencing

Two biological replicates were analyzed of: HD180 iPSCs, HD180 iBMECs, HD-corrected iPSCs, and HD-corrected iBMECs. Given recent guidelines for RNA sequencing which suggest a minimum of three replicates [18], a limitation is that our analysis may be underpowered. iPSCs were harvested prior to differentiation in UM/F- media, while iBMECs were harvested as confluent monolayers 2 days following sub-culture on collagen IV and fibronectin-coated tissue-culture plates. To harvest total RNA, cells were lysed using RLT buffer supplemented with β-mercaptoethanol and then RNA isolated using a RNeasy Mini Kit with DNase I digestion (Qiagen #79254). All sequenced samples had RNA integrity numbers above 9.7, as measured by an Agilent 2100 bioanalyzer. Total RNA was subjected to oligo (dT) capture and enrichment, and the resulting mRNA fraction was used to construct cDNA libraries. Approximately 20 million paired end 150 bp reads were collected per sample using Illumina NovoSeq (performed by Novogene). Alignment and quantification to reference genome (GRCh38) was performed using *Rsubread* (v2.0.1) [19]. Transcript abundances are presented as fragments per kilobase of transcript per million mapped reads (FPKM). Normalization (rlog transformed), visualization, and differential analysis was performed using *DESeq2* (v1.28.1) [20]. Differentially expressed genes (DEGs) were determined using the Wald test with Benjamini–Hochberg correction (adjusted *p* values < 0.05 was considered statistically significant). Pathway enrichment analysis (Hallmark gene sets and GO biological processes terms) was conducted on DEGs using Enrichr, with built in statistical analysis used at a adjusted p-value cutoff of 0.05 [21]. RNA-seq data have been deposited in GEO under accession number GSE194416.

### Barrier function measurements

Transendothelial electrical resistance (TEER; Ω cm^2^) was recorded (World Precision Instruments #EVOM2) as previously reported [16]. Measurements were performed on 6.5 mm Transwells with a 0.4 μm pore size polyester membrane insert (Corning #CLS3470). TEER values were corrected for the resistance of the Transwell insert without cells. iBMECs were seeded at a density of 0.33–1.00 × 10^6^ cm^−2^ onto Transwells in endothelial media as previously described. After 24 h, medium was switched to basal medium and daily recordings were collected for 10 days without additional media switches. To reduce temperature-dependent effects, TEER values were recorded within 1 min following removal from the incubator, alternating measurements between experimental conditions.

At day two, the permeability of 200 μM Lucifer yellow (ThermoFisher #L453), 2 μM AlexaFluor647-conjugated 10 kDa dextran (ThermoFisher #D22914), 10 μM rhodamine 123 (ThermoFisher #R302), and 25 mM D-glucose (Sigma #G8270) across BMEC monolayers was measured using previously reported protocols [16]. A subset of permeability experiments were also performed on day 10. The following excitation and emission settings were utilized on a Synergy™ H4 microplate reader (Biotek): Lucifer yellow (428 nm/545 nm), 10 kDa dextran (647 nm/667 nm), rhodamine 123 (495 nm/525 nm). Glucose transport was quantified using a colorimetric detection kit (ThermoFisher #EIAGLUC) following the manufacturer’s protocol and absorbance measurements were performed at 560 nm. Concentrations were determined from calibration curves based on serial dilution of each compound spanning four orders of magnitude. The apparent permeability of each compound was calculated as *P* = *(dC/dt)(V)(1/A)(1/C*_*0*_*)*, where *dC/dt* is the slope of cumulative concentration, *V* is the volume of the receiving compartment (i.e. basolateral or apical chamber), *A* is the area of the monolayer, and *C*_*0*_ is the dosed concentration of solute [22]. For rhodamine 123, efflux ratios were calculated as the ratio of basolateral-to-apical and apical-to-basolateral permeability normalized to 10 kDa dextran (a non-efflux substrate). To ensure that measurements were not limited by transport across the porous membrane, we confirmed that permeability values for Lucifer yellow and glucose were more than ten-fold lower than permeabilities in Transwells with no cells. Biological replicates of permeability measurements were averaged across at least two Transwells (technical replicates).

### Responsiveness to chemical perturbation

A bead angiogenesis assay was conducted as previously reported [23]. Briefly, 150 μm diameter Cytodex™ 3 microcarrier beads (Sigma #C3275) were coated with collagen IV and fibronectin and then seeded with singularized iBMECs in endothelial media supplemented with 1% penicillin–streptomycin and 10 μM ROCK inhibitor. After 80 min, with gentle agitation every 20 min by slowly pipetting 1 mL of fresh medium onto settled beads, the beads were washed to remove non-adherent cells and then cultured on a shaker at 100 rpm for 24 h. Next, beads were embedded within 6 mg mL^−1^ neutralized rat tail type I collagen (Corning #354249) and treated with basal media with and without 50 ng mL^−1^ recombinant human VEGF-165 (VEGF; Biolegend #583704). After 3 days in culture, the sprout density (# bead^−1^) was manually counted from phase contrast images across at least 8 beads (technical replicates) for each condition.

For oxidative and osmotic stress experiments, iBMECs were seeded onto Transwells as previously described and exposed to hydrogen peroxide (H_2_O_2_; Sigma #H1109) or mannitol (Sigma #M4125) after 48 h. To avoid the need for a medium switch, 5 μL of concentrated H_2_O_2_ freshly prepared in sterile water was added to the apical chamber of Transwells and gently mixed by pipetting to achieve final concentrations of 0.2 to 1 mM. TEER was recorded daily after exposure without medium switches being conducted. As mannitol induces BBB opening near its concentration limit, the medium was changed to basal medium with 1.4 M mannitol for 10 min, and then switched to basal medium. TEER was recorded immediately before treatment, immediately after treatment, 1 h later, and 1 day later. To visualize reactive oxygen species and actin cytoskeleton, some Transwells were treated with 50 μM CellROX® Green Reagent (Invitrogen #C10444), AlexaFluor647 phalloidin (ThermoFisher #A22287), and DAPI solution for 30 min at 37 °C after 1 day of exposure to 0.6 mM H_2_O_2_.

### Tissue-engineered BBB microvessels

Tissue-engineered BBB microvessels were fabricated as previously reported [24]. iBMECs were sub-cultured for 1 h and then detached using Accutase before seeding into 150 μm diameter channels patterned in 7 mg mL^−1^ type I collagen. Prior to seeding, the collagen matrix was cross-linked with 20 mM genipin (Wako #078-03021) to increase stiffness and then the channel surface was coated in collagen IV and fibronectin to promote cell adhesion. Cells were seeded into microvessels and cultured for 30 min under no flow to facilitate adhesion, then microvessels were perfused at ~ 1 dyne cm^−2^ shear stress for the remainder of experimentation. After formation of confluent monolayers, microvessels were perfused with 200 μM Lucifer yellow for 1 h, with images collected every 2 min. The permeability was calculated from a plot of fluorescence versus time, as previously reported [25]. Microvessels were imaged using a 10× objective (Nikon) with epifluorescence illumination provided by an X-Cite 120LED-Boost (Excelitas Technologies). The turnover of iBMECs in microvessels was calculated from phase contrast images acquired simultaneously with fluorescence images. Cell loss and cell proliferation events were manually counted on the top plane of the microvessel as previously reported [24]. From counts of cell loss and proliferation events, values were normalized to total number of cells in the imaging plane and to time, with units of % h^−1^. Net microvessel turnover was calculated as the difference in the rates of proliferation and loss (% h^−1^).

THP-1 (ATCC® TIB-202™) is a human leukemia monocytic cell line [26]. THP-1 s were grown in suspension with RPMI-1640 Medium (ThermoFisher #11875093) supplemented with 10% fetal bovine serum (Sigma #F4135) and 1% penicillin–streptomycin. Before use, cells were labeled with 5 μM Calcein AM (ThermoFisher #C3100MP) for 15 min, and then resuspended at 1 × 10^6^ cells mL^−1^ in basal media. Microvessels were perfused with THP-1 s under low shear stress (~ 0.2 dyne cm^−2^) for 10 min, and then washed out using higher shear stress (~ 2 dyne cm^−2^). Adherent immune cells were manually counted using fluorescence microscopy.

### Statistical analysis

Statistical analysis was performed using Prism ver. 8 (GraphPad) with metrics presented as mean ± SEM (standard error of the mean). A student’s unpaired t-test (two-tailed with unequal variance) was used for comparison of two groups. A two-way ANOVA with analysis of interaction and Bonferroni’s multiple comparisons test was used for comparison of angiogenic response. The number of biological replicates are reported in figure legends. Differences were considered statistically significant for *p* < 0.05, with thresholds of *p < 0.05, **p < 0.01, and ***p < 0.001.

## Results

### hiPSCs with elevated CAG repeats display a unique BMEC differentiation trajectory

**Fig. 1:**
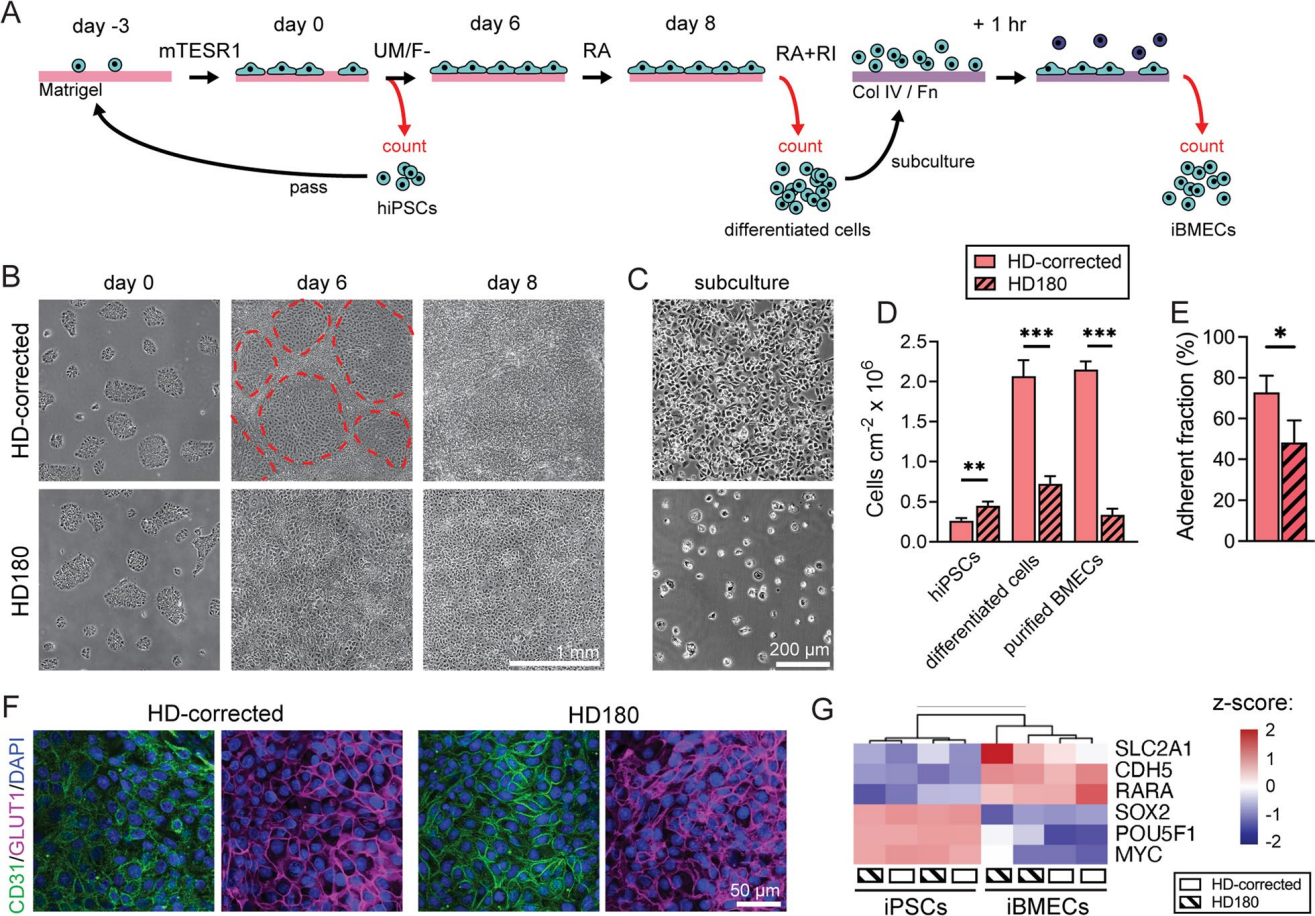
Comparison of differentiation of HD-corrected and HD180 iPSCs reveals a unique iBMEC differentiation trajectory. **A** Schematic illustration of differentiation timeline. hiPSC colonies are counted and passaged at 10,000 cells cm^−2^ on Matrigel-coated plates. iBMECs are differentiated over 8 days (six-day treatment with UM/F- media and two-day treatment with RA media). **B** Representative phase contrast images of differentiation at day 0, 6, and 8 for HD-corrected and HD180 iPSCs. Endothelial colonies surrounded by neural tracts only form during HD-corrected iBMEC differentiation (red dotted line), despite identical density and appearance of iPSC colonies between the two iPSCs. **C** Representative phase contrast images following sub-culture of HD-corrected and HD180 differentiated cells highlights the differences in fraction of adherent cells. **D** Cell density on day 0 (hiPSCs 3 days after passing at 10,000 cm^−2^), day 8 (differentiated cells), and post-subculture (purified iBMECs). Data collected across *n* = 7 and 8 independent differentiations for each cell line, respectively. **E** iBMEC adherent fraction (ratio of adherent cells to differentiated cells) for HD-corrected and HD180 iBMECs. Data collected across *n* = 4 and 5 independent differentiations for each cell line, respectively. **F** Representative immunofluorescence images of CD31 and GLUT-1 for HD-corrected and HD180 iBMECs. **G** iBMEC differentiation downregulates genes associated with pluripotency (*POU5F1*, *SOX2*, *MYC*) independent of CAG repeat length, and upregulates genes associated with endothelial and BMEC phenotype (*CDH5*, *SLC2A1*, *RARA*). Data represents row z-scores for transformed bulk RNA sequencing data across *n* = 2 independent differentiations for HD-corrected and HD180 cells. Transcript abundances (FPKM values) shown in Additional file 2: Fig. S1A

Brain microvascular endothelial-like cells (iBMECs) were differentiated from an isogenic pair of iPSCs with 180 (HD180) and 18 (HD-corrected) CAG repeats in the *HTT* gene. Differentiation was conducted by sequential treatment with mTeSR1 for 3 days, UM/F- for 6 days, and endothelial media (RA) for 2 days, as previously reported [15, 16] (Fig. 1A). Next, cells were purified by sub-culture onto collagen IV and fibronectin-coated plates and detached for seeding onto glass, Transwells, or tissue-engineered microvessels. The differentiation of the HD180 iPSCs was visibly unique compared to isogenic HD-corrected iPSCs and other control iPSCs; while HD180 iPSC colonies appeared similar, treatment with UM/F- resulted in limited neural tracts, which is a hallmark of iBMEC differentiation (dotted red line; Fig. 1B). Additionally, following sub-culture to selectively purify iBMECs, differentiation of HD180 iPSCs produced significantly fewer adherent cells compared to the isogenic control (Fig. 1C). Cell counting during differentiation showed a higher density of HD180 iPSCs after the three days in mTeSR1 (*p* = 0.005) (Fig. 1D). In contrast, following differentiation and sub-culture we observed a higher density of HD-corrected cells (*p* < 0.001 for both comparisons) (Fig. 1D). Additionally, the adherent fraction of the differentiation (defined as the number of adherent cells following sub-culture divided by the number of differentiated cells) was lower for the HD180 iPSC differentiation (*p* = 0.038) (Fig. 1E). These results suggest a unique differentiation trajectory for iPSCs with expanded CAG repeats. However, adherent cells from both iPSC sources displayed uniform immunoreactivity for platelet endothelial cell adhesion molecule (CD31) and glucose-transporter 1 (GLUT-1) (Fig. 1F). Additionally, across both differentiations there was a loss in gene expression of pluripotency markers (*POU5F1*, *SOX2*, *MYC*) and gain in gene expression of VE-cadherin (*CDH5*), GLUT-1 (*SLC2A1*), and retinoic acid receptor alpha (*RARA*) (Fig. 1G, Additional file 2: Fig. S1A). CD31, VE-cadherin, and GLUT-1 expression have been utilized to assess differentiation efficiency [5, 27], while *RARA* upregulation was previously shown to induce barrier function following iBMEC differentiation [28]. Together these results suggest that independent of CAG expansion, the differentiation produced brain endothelial-like cells.

### CAG expansion alters barrier function

**Fig. 2:**
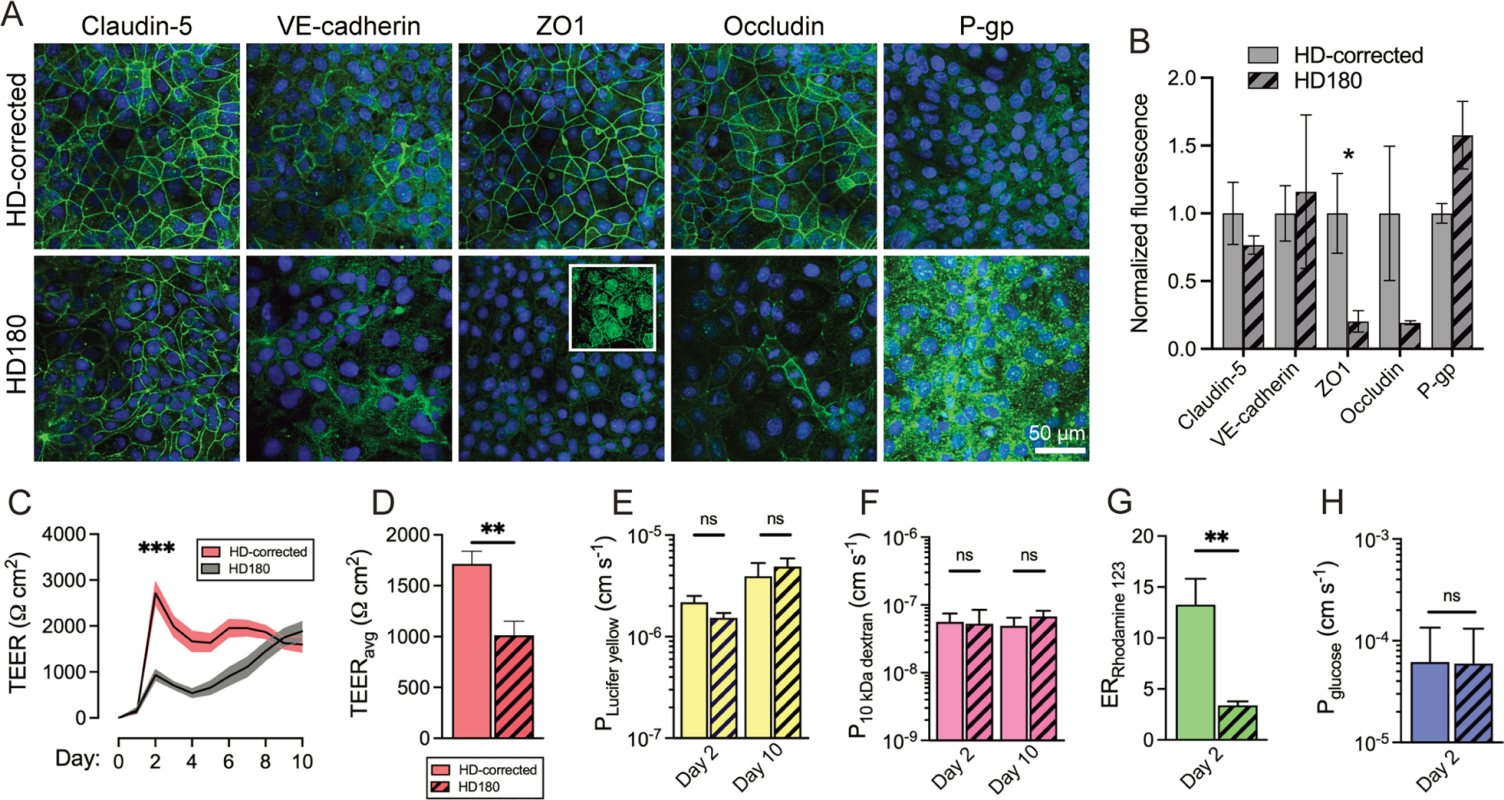
HD180 iBMECs exhibit altered protein localization, gene expression*,* and BBB function. **A** Immunofluorescence of BMEC proteins (claudin-5, VE-cadherin, ZO1, occludin, ZO1, P-glycoprotein). Representative images of iBMECs are shown at 2 days after seeding. Inset shows mislocalization of ZO-1 at higher magnification for HD180 iBMECs. **B** Semi-quantitative analysis of iBMEC protein expression at day 2 following differentiation. The fluorescence signal was normalized to the nuclear DAPI signal and then plotted relative to HD-corrected. Data collected across *n* = 4–6 independent differentiations. **C**, **D** TEER time course and average over 10 days. Data collected across *n* = 16 (HD-corrected) and 26 (HD180) independent differentiations. **E**, **F** Lucifer yellow and 10 kDa dextran permeability (day 2 and day 10). Data collected across *n* = 6 (HD-corrected) and 8 (HD180) independent differentiations. **G** Rhodamine 123 efflux ratio (day 2). Data collected across *n* = 4–5 independent differentiations for HD-corrected and HD180 iBMECs. **H** Glucose permeability (day 2). Data collected across *n* = 4 independent differentiations for HD-corrected and HD180 iBMECs. All recordings in **C**–**G** represent averages across *n* = 2–6 technical replicates (individual Transwells) for each biological replicate

We characterized iBMEC monolayers via immunofluorescence imaging of BMEC markers and functional measurements of transendothelial electrical resistance (TEER) and permeability (Fig. 2). Monolayers of HD180 iBMECs displayed similar staining of claudin-5 and VE-cadherin, decreased staining of occludin and zonula occludens-1 (ZO1), and slightly increased staining of P-gp compared to HD-corrected iBMECs (Fig. 2A). We conducted semi-quantitative analysis of fluorescence intensity normalized to the nuclear signal, finding that only ZO-1 was significantly reduced in HD180 iBMECs (*p* = 0.040) (Fig. 2B). In HD180 iBMECs, the ZO-1 signal was poorly localized to junctions, with substantial signal in the nucleus (Fig. 2A-inset).

The HD-corrected cells exhibited a typical TEER time course [15], with peak values of ~ 3000 Ω cm^2^ on day two followed by a gradual decrease. The HD180 cells exhibited a unique trajectory, with ~ three-fold lower TEER on day 2 (*p* < 0.001). Both HD180s and HD-corrected iBMECs exhibited TEER values > 1000 Ω cm^2^ from days 6–10 (Fig. 2C). The average TEER over 10 days was ~ 1024 Ω cm^2^ and ~ 2067 Ω cm^2^ for HD180s and HD-corrected iBMECs, respectively (*p* < 0.001) (Fig. 2D). Despite differences in TEER, the permeability of Lucifer yellow (444 Da) in Transwells was independent of CAG expansion at both day two (*p* = 0.171) and day ten (*p* = 0.606) (Fig. 2E), indicating preserved paracellular permeability despite CAG expansion.

Permeability measurements with 10 kDa dextran matched observations with Lucifer yellow (Fig. 2F). The Lucifer yellow permeability was ~ 2 × 10^–6^ cm s^−1^ on day two and ~ 4 × 10^–6^ cm s^−1^ on day ten, while the permeability of 10 kDa dextran was ~ 6 × 10^–8^ cm s^−1^ on days 2 and 10. These values match previous measurements of iBMEC permeability across other iPSC sources [16]. We also measured the efflux ratio (ratio of apical-to-basolateral to basolateral-to-apical permeability) of rhodamine 123 (R123), a substrate of the P-gp efflux pump. HD180 iBMECs exhibited lower efflux ratios for R123 compared to HD-corrected cells (*p* = 0.005) (Fig. 2G). While not statistically significant (*p* = 0.125), the apical-to-basolateral permeability of R123 was ~ three-fold higher for HD180 iBMECs; these results suggest that the reduced efflux ratio could result from both improper polarization and reduced activity, which could be further explored using P-gp inhibition. Both HD-corrected and HD180 iBMECs displayed similar glucose permeability (*p* = 0.970) (Fig. 2H).

We conducted additional analysis of immunofluorescence images at day 10, finding similar expression of claudin-5 and VE-cadherin between HD-corrected and HD180 iBMECs (Additional file 2: Fig. S2). However, the VE-cadherin signal was particularly diminished at day 10 compared to day 2, suggesting a loss of endothelial phenotype during extended culture. For this reason, subsequent functional assays were conducted at day 2, which corresponds to iBMECs displaying peak TEER values and localized adherens and tight junctions. Additionally, we did not observe aggregates of mutant HTT within iBMECs, independent of CAG expansion: the mEM48 fluorescence signal was similarly negligible for both cell sources (*p* = 0.857) (Additional file 2: Fig. S2A, B).

### Changes in barrier function are independent of differentiation variables

iBMEC differentiation is sensitive to variables including reagent source, seeding density, and serum lot [17, 29, 30]. Therefore, we sought to determine whether differentiation variables would alter differences in phenotype between HD-corrected and HD180 iBMECs. We tested the effects of iPSC seeding density, Transwell seeding density, media volume, and use of serum free alternatives.

After seeding iPSCs at 5000, 10,000, 20,000, 30,000, and 40,000 cells cm^−2^ we counted viable cells post-mTeSR1, post-RA, and post-subculture (Fig. 1A). After incubation in mTeSR1 (before starting the iBMEC differentiation), HD180 and HD-corrected cells showed similar iPSC cell counts at high initial seeding densities (> 10,000 cells cm^−2^), while HD180 iPSCs were more populous at lower seeding densities (**≤ **10,000 cells cm^−2^) (*p* < 0.01 for both comparisons) (Additional file 2: Fig. S3A). Post-differentiation, the HD180 line produced significantly fewer cells before (post-RA) and after sub-culture, regardless of the initial seeding density (*p* < 0.01 for all comparisons) (Additional file 2: Fig. S3B, C). The adherent fraction was maximized at lower initial seeding densities (5000 and 10,000 cells cm^−2^), although the adherent fraction for HD180 cells was significantly lower (*p* < 0.05) (Additional file 2: Fig. S3D).

Across all initial seeding densities and both cell sources, we seeded Transwells directly with 0.33 × 10^6^ and 1.0 × 10^6^ cells cm^−2^. In addition, we performed a sub-culture purification prior to seeding Transwells at 0.33 × 10^6^ cells cm^−2^. Statistically significant differences in TEER were observed across all different seeding approaches at the initial seeding density of 10,000 cells cm^−2^ (*p* < 0.05 for all comparisons) (Additional file 2: Fig. S4). We also examined the effect of increased medium volume during the differentiation and the use of the serum replacement B-27 (Additional file 2: Fig. S5). We found that performing the differentiation in 2 mL of medium decreased the average TEER significantly for the HD180 (*p* = 0.013) but not significantly for HD-corrected cells (*p* = 0.247). Interestingly, the use of a serum-free differentiation decreased average TEER, contrary to previous reports [17]; the average TEER was significantly lower in the HD180 cells (*p* = 0.021) but not significantly lower in the HD-corrected cells (*p* = 0.352).

### Non-isogenic HD iBMECs also maintain paracellular barrier

In healthy individuals the average *HTT* gene CAG repeat length is 20 [31]. To compare changes in iBMEC phenotype across a broader range of CAG repeat lengths, we tested barrier function of two additional non-isogenic iPSCs: (1) an adult-onset HD iPSC line with 50 CAG repeats (HD50), and (2) a control iPSC line with 21 CAG repeats (HD21) (Additional file 2: Table S1). Differentiations were conducted matching the optimized protocol for HD180 and HD-corrected iPSCs (i.e. initial seeding density of 10,000 cells cm^−2^, subculturing to purify cells, and seeding on Transwells at 0.33 × 10^6^ cells cm^−2^). The fraction of adherent iBMECs was CAG-length dependent across all iPSCs (Additional file 2: Fig. S6A). Similarly, TEER values were higher for the lower CAG lengths (18 and 21), although not statistically significant (Additional file 2: Fig. S6B). Despite general reductions in TEER observed in HD iBMECs, our results suggest that paracellular barrier function is maintained in HD iBMECs, as TEER values remain higher than previously reported values at similar CAG repeat lengths [5].

### CAG expansion uniquely alters gene expression of HD iBMECs

**Fig. 3:**
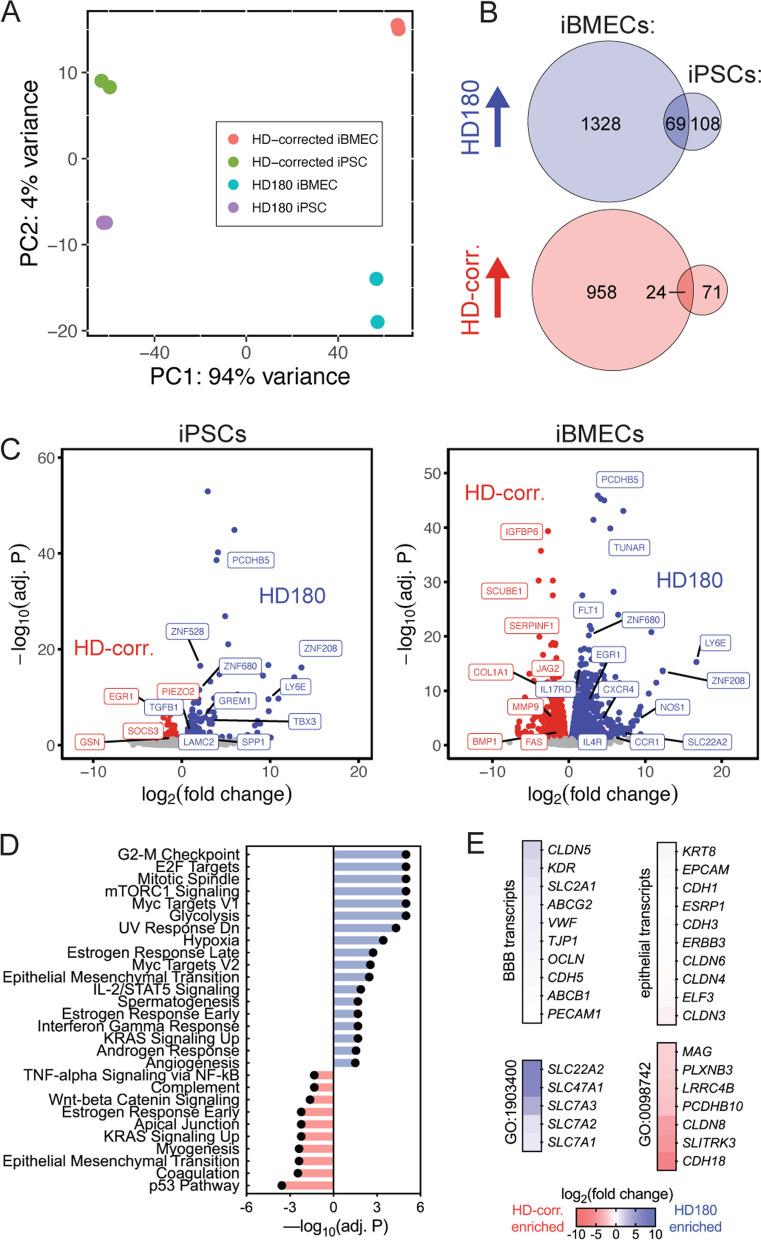
HD180 iBMECs exhibit unique gene expression profiles. **A** Principal component analysis of bulk RNA sequencing. Data collected across *n* = 2 independent differentiations for each cell line, where iPSC and iBMEC RNA is paired. **B** Venn diagrams showing overlap of up and downregulated CAG expansion-dependent genes in iBMECs versus iPSCs. **C** Volcano plots comparing gene expression of iPSC and iBMECs from HD180 (blue) and HD-corrected (red) sources. Selected up and downregulated genes are labeled. **D** Hallmark gene sets from the Molecular Signatures Database (MSigDB) that are significantly enriched (blue) or depleted (red) in HD180 compared to HD-corrected iBMECs. Significance truncated for gene sets with –log_10_(adj. P) > 5. **E** Heatmaps of endothelial, epithelial, or GO term transcript changes between HD180 iBMECs and HD-corrected iBMECs

Huntington’s disease results in widespread transcriptional dysregulation in the brain [32–34]. Bulk RNA sequencing was utilized to compare global gene expression profiles between HD180 and HD-corrected iPSCs, and the corresponding HD180 and HD-corrected iBMECs (Fig. 3). Principal component analysis (PCA) showed that distinct gene expression profiles predominately emerged following iBMEC differentiation (Fig. 3A). Less than 5% of up and downregulated genes were shared between iPSCs and iBMECs, suggesting a distinct impact of CAG expansion on the two cell types (Fig. 3B).

We identified 177 upregulated and 95 downregulated genes between HD180 and HD-corrected iPSCs; however, gene set enrichment analysis (GSEA) using hallmark gene sets from the Molecular Signatures Database (MSigDB) found that only a single gene set was significantly enriched (Epithelial Mesenchymal Transition; enriched in HD180 iPSCs) (Fig. 3C). In contrast, we identified 1397 upregulated and 982 downregulated genes between HD180 and HD-corrected iBMECs, corresponding to 18 enriched and 10 depleted hallmark gene sets in HD180 iBMECs (Fig. 3C). Enriched hallmark gene sets for HD180 iBMECs included those involved in cell growth and division (G2-M checkpoint, mitotic spindle), hypoxia, immune response (IL-2/STAT5 signaling, interferon gamma response), and angiogenesis, whereas depleted hallmark gene sets included those involved in canonical brain endothelial functions including coagulation, apical junction, Wnt/beta-catenin signaling, tumor necrosis factor (TNF)-α signaling, and complement (Fig. 3D). Notable differentially expressed genes, including those driving enrichment of hallmark gene sets are labeled in Fig. 3C, including *IL4R, CCR1*, *SLC22A2*, *NOS1*, *CXCR4*, *IL17RD*, *EGR1*, *ZNF208*, *LY6E*, *ZNF680*, *FLT1*, *TUNAR*, and *PCDHB5* in HD180 iBMECs, and *FAS*, *BMP1*, *MMP9*, *COL1A1*, *JAG2*, *SERPINF1*, *SCUBE1*, and *IGFBP6* in HD-corrected iBMECs. Additional file 1 has complete results of transcript abundances across experimental groups and differential gene expression analysis.

Specific gene sets were explored corresponding to canonical BBB transcripts, epithelial transcripts, and enriched GO terms (Fig. 3E). Canonical BBB genes were not differentially expressed between HD-corrected and HD180 iBMECs (Fig. 3E, Additional file 2: Fig. S1B), in contrast to the observation of mislocalized tight junction proteins from immunofluorescence studies. Recent work has shown that iBMECs possess epithelial characteristics [35]. We found that iBMEC differentiation for both HD180 and HD-corrected iPSCs was associated with upregulation of some epithelial markers (Additional file 2: Fig. S1A), however, both HD-corrected and HD180 iBMECs displayed similar epithelial transcript abundances (Fig. 3E). This crucially suggests that differences in epithelial identity do not drive phenotype differences between HD-corrected and HD180 iBMECs. The most enriched GO term in HD180 iBMECs was l-arginine transmembrane transport (GO:1903400), while the most enriched GO term in HD-corrected iBMECs was cell–cell adhesion via plasma-membrane adhesion molecules (GO:0098742). The corresponding transcripts were highly differentially expressed (Fig. 3E), and may suggest increased L-arginine transport and compromised cell–cell adhesion due to expanded CAG repeats in iBMECs.

### Responsiveness to oxidative, angiogenic, and osmotic stimuli

**Fig. 4:**
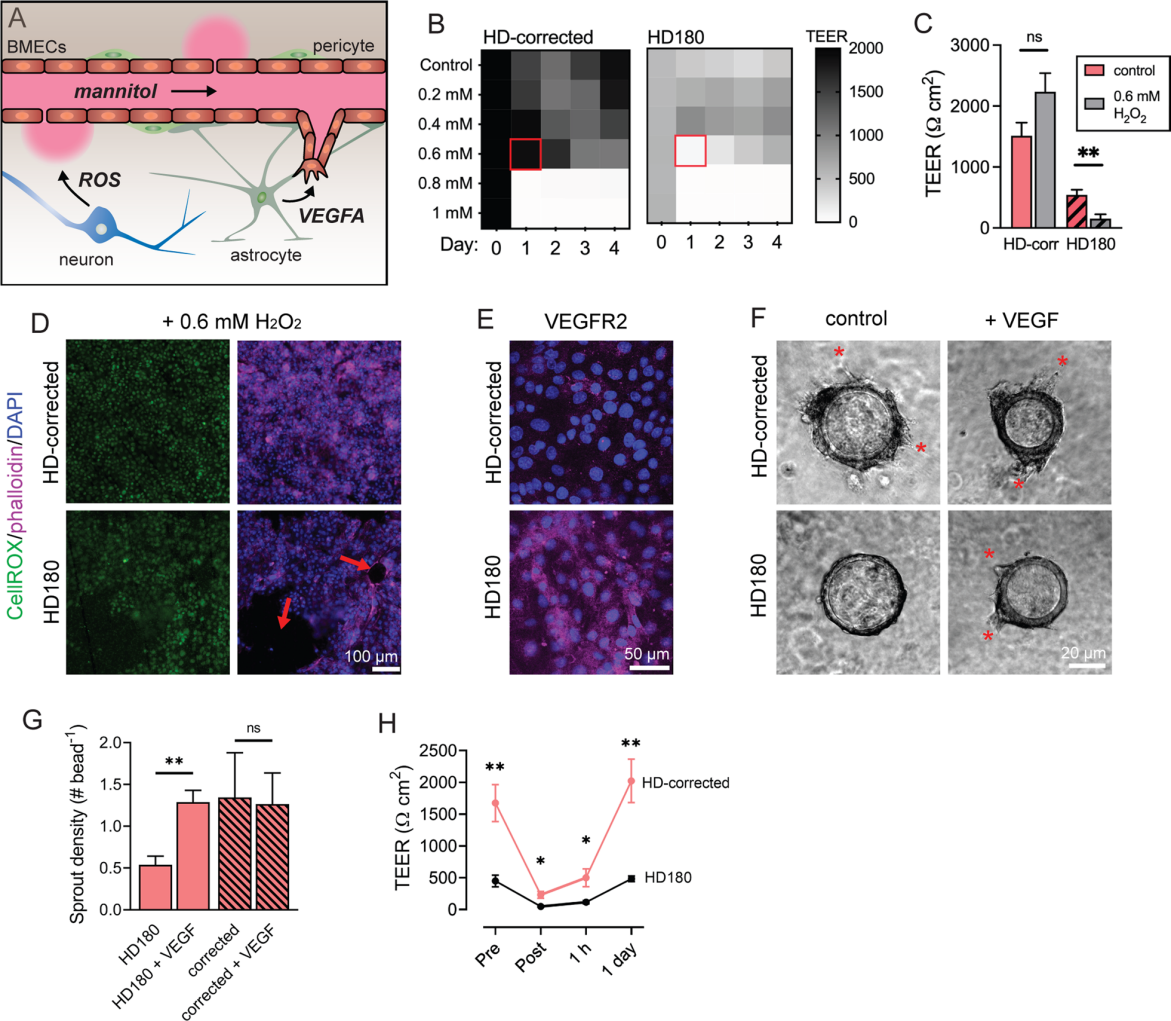
HD180 iBMECs show unique responses to oxidative, angiogenic, and osmotic stress. **A** Schematic illustration of disease- and therapeutic-relevant perturbations to the HD BBB. **B**–**D** HD180 iBMECs are more vulnerable to oxidative damage: **B** time course of iBMEC TEER in response to various H_2_O_2_ concentrations. Red box denotes concentration resulting in most unique responses between cell sources. **C** iBMEC TEER 24 h after exposure to 0.6 mM H_2_O_2_. Data collected across *n* = 7 (HD180) and 6 (HD-corrected) independent differentiations. **D** Representative fluorescence images of cellular reactive oxygen species, nuclei, and f-actin (Phalloidin) 24 h after exposure to 0.6 mM. Red arrows indicate holes in endothelium. **E** Representative immunofluorescence images of VEGFR2. Data collected across *n* = 6 (HD180) and 4 (HD-corrected) independent differentiations. Quantification shown in Additional file 2: Fig. S2C. **F** Bead angiogenesis assay. Beads coated in iBMECs seeded in 6 mg mL collagen I + Matrigel, then supplemented with basal media or with 20 ng mL^−1^ bFGF and 50 ng mL^−1^ VEGF. Representative images show beads 72 h after treatment, where red asterisks denote angiogenic sprouts. **G** Quantification of sprout density across bead angiogenesis assay conditions. Data collected across *n* = 4 (HD180) and 3 (HD-corrected) independent differentiations. **H** Changes in TEER in response to osmotic treatment (10 min exposure to 1.4 M mannitol). Data collected across *n* = 5 independent differentiations

We sought to determine how HD iBMECs responded to pathological perturbations, given transcriptional dysregulation of many cellular processes associated with HD disease progression. To do so, we assessed the response of HD180 iBMECs to oxidative, angiogenic, and osmotic stress (Fig. 4A). Increased oxidative stress markers are detected in peripheral blood of HD patients and asymptomatic HD gene carriers [36–38]. Previous studies have found that the iPSC-derived microglia and neurons harboring expanded CAG repeats release elevated levels of ROS and are hypersensitive to exogenous stress [39, 40]. We evaluated the effect of oxidative stress on iBMECs exposed to a range of H_2_O_2_ concentrations (0.2–1 mM) by measuring TEER [41]. H_2_O_2_ can exert concentration-dependent effects on BMEC phenotype, including induction of apoptosis or angiogenesis [23, 42]. There was a sharp decrease in TEER values at an H_2_O_2_ concentration greater than 0.6 mM for both HD-corrected and HD180 iBMECs (Fig. 4B). This concentration is within the range where pathological effects are observed following inhalation or ingestion [43]. Following exposure to 0.6 mM H_2_O_2_ for 24 h, HD180 iBMECs showed dramatically reduced TEER compared to vehicle (~ 150 Ω cm^2^) (*p* = 0.008) (Fig. 4B). In contrast, there was no statistical difference in TEER between 0.6 mM H_2_O_2_ and vehicle for HD-corrected iBMECs (*p* = 0.095) (Fig. 4C). Staining of HD180 iBMECs exposed to 0.6 mM H_2_O_2_ revealed gaps in the monolayer, whereas HD-corrected monolayers remained intact (Fig. 4D).

Post-mortem HD tissue is characterized by an increase in angiogenic microvessels [3, 4], while increased astrocytic secretion of VEGF-A is observed in HD mouse models (R6/2) [8]. An increased angiogenic phenotype has also been inferred from an in vitro wound healing assay in HD-iBMEC monolayers [5]. To test angiogenic potential, we performed a bead angiogenesis assay by coating 150 μm diameter beads with iBMECs [23]. After formation of a confluent monolayer, the beads were embedded within a collagen I and Matrigel matrix, and then exposed to 50 ng mL^−1^ vascular endothelial growth factor (VEGF). HD180 iBMECs displayed ~ two-fold elevated VEGFR2 protein expression compared to HD-corrected cells (*p* = 0.049) (Fig. 4E, Additional file 2: Fig. S2C); a similar fold difference was observed at the transcriptional level (*KDR*), but was not statistically significant. Sprout density increased in response to VEGF treatment for HD180 iBMECs (*p* = 0.003), but was unchanged for HD-corrected iBMECs (*p* = 0.999) (Fig. 4F,G). We also measured relative angiogenic activity as the percentage of all imaged beads that displayed visible sprouts; the percentage of angiogenic beads increased in response to VEGF treatment for HD180 iBMECs (*p* = 0.023), but was unchanged for HD-corrected iBMECs (*p* = 0.280). These results suggest that for short durations of VEGF exposure (72 h), HD180 iBMECs display unique angiogenic responsiveness.

While recent therapeutic approaches for HD utilize intrathecal delivery to bypass the BBB, BBB opening (BBBO) represents a possible strategy to increase drug delivery to neurons following intravenous delivery. Osmotic BBBO utilizes intra-arterial infusion of hyperosmotic agents to transiently disrupt cell–cell junctions thereby enabling delivery of large molecular weight compounds into the brain [44, 45]. We hypothesized that HD iBMECs may have unique responses to osmotic stress, given a recent report that AD iBMECs (*PSEN1* mutations) displayed altered responsiveness to focused ultrasound (FUS), another strategy for transient BBBO [46]. To test osmotic stress response, we treated iBMEC monolayers with clinical concentrations of the hyperosmotic agent mannitol (1.4 M), used for osmotic BBBO, for 10 min. HD180 iBMECs displayed lower TEER values that HD-corrected iBMECs immediately following mannitol treatment (*p* = 0.011) and 1 h later (*p* = 0.029). While unsurprising given the initial lower TEER values of HD iBMECs (pre-mannitol), our results suggest further weakened paracellular barrier in response to osmotic stress. This TEER difference would be expected to result in lower Lucifer yellow permeability for HD180 iBMECs given our previous studies identifying an inverse relationship between TEER and permeability below 250 Ω cm^2^ [16].

### Tissue-engineered HD BBB microvessels

**Fig. 5:**
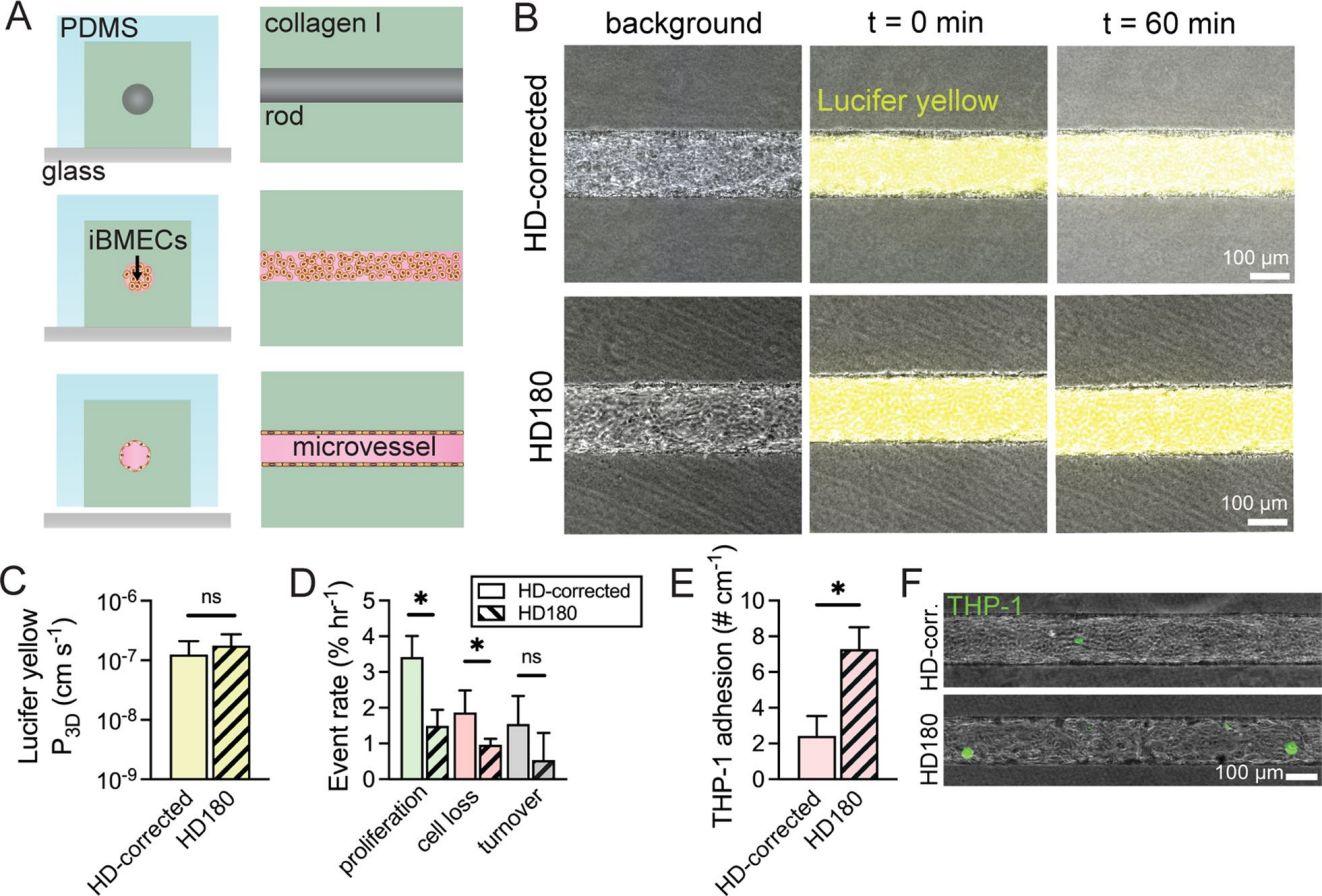
Tissue-engineered BBB microvessel model incorporating HD180 or HD-corrected iBMECs. **A** Schematic illustration of fabrication of three-dimensional microvessels seeded with iBMECs; (left) front view, (right) side view. **B** Microvessels with HD-corrected and HD180 iBMECs similarly restrict Lucifer yellow transport. Representative images are shown. **C** Lucifer yellow permeability in 3D microvessels. Data collected across *n* = 5 (HD-corrected) and 4 (HD180 iBMECs) independent differentiations. **D** Turnover rates of microvessels. Data collected across *n* = 5 (HD-corrected) and 4 (HD180 iBMECs) independent differentiations. **E**, **F** Adhesion of monocyte-like cells to tissue-engineered microvessels: **E** Data collected across *n* = 4 (HD-corrected) and 3 (HD180) independent differentiations of iBMECs seeded into microvessels. **F** Representative images of adherent cells after washout. THP-1 fluorescence is oversaturated to assist in visualization

To study barrier function and endothelial cell turnover in real-time we generated three-dimensional tissue-engineered microvessels, as previously reported (Fig. 5A) [24]. Tissue-engineered models recapitulate many microenvironmental cues present in the human cerebrovasculature (i.e. shear stress and cell-ECM interactions) [47]. Similar to results in Transwells, we found that the permeability of Lucifer yellow in HD180 microvessels was identical to the value in HD-corrected microvessels (*p* = 0.691) (Fig. 5B, C). However, Lucifer yellow permeability was ~ ten-fold lower in 3D microvessels compared to 2D Transwell measurements, as also noted previously using a different iPSC source [16]. To assess endothelial cell dynamics in BBB microvessels with HD180 or HD-corrected iBMECs, the rates of proliferation and cell loss were tracked from time lapse phase contrast imaging acquired during permeability measurements (Fig. 5D). HD180 microvessels showed ~ two-fold lower rates of proliferation (*p* = 0.042) and cell loss (*p* = 0.026) compared to microvessels formed from HD-corrected cells. These results suggest that HD180 microvessels display unique dynamics of endothelial turnover. Additionally, based on findings of altered innate immune responses from GSEA, we measured the adhesion of monocyte-like cells (THP-1 s) in iBMEC microvessels. We found that HD180 microvessels displayed elevated adhesion of immune cells compared to microvessels formed from HD-corrected cells (*p* = 0.033) (Fig. 5E). There was an ~ three-fold increase in adhesion despite lack of external inflammatory stimuli (e.g. TNF-α), suggesting that HD180 iBMECs display activated innate immune response. Interestingly, the transcript abundance and immunofluorescence intensities of ICAM-1 and VCAM-1, two critical surface adhesion molecules for leukocyte trafficking, were similar between HD-corrected and HD180 iBMECs in 2D (Additional file 2: Figs. S1B, S2C). Thus, increased adhesion of THP-1s on HD180 iBMECs is likely mediated by other differences in gene/protein expression or is dependent on 3D microenvironment (where gene/protein expression is distinct [48]).

## Discussion

### Summary of changes in BMEC phenotype

Comparison of HD180 to HD-corrected iBMECs (using an optimized differentiation protocol) revealed the following key results: (1) a reduction in TEER but no difference in permeability, (2) a reduction in efflux activity, (3) transcriptional dysregulation, (4) decreased endothelial cell loss and proliferation, (5) unique responses to oxidative and osmotic stress, (6) increased responsiveness to VEGF (and elevated expression of *VEGFR2*), and (7) increased immune cell adhesion. For some of these findings, we identified discrepancies between gene and protein expression. For example, while tight junction transcripts were similarly expressed, HD180 iBMECs displayed mislocalized ZO-1 and reduced TEER. Overall, our results imply that paracellular barrier function of BMECs is likely maintained in juvenile HD, while BMECs may be increasingly vulnerable to pathological perturbations. Additionally, CAG length may modulate the severity of changes in iBMEC phenotype, matching findings that CAG repeat length is associated with the age of HD onset [49].

### Differentiation

Although differentiation of HD-corrected iPSCs was similar to other iPSCs from healthy individuals (e.g. formation of neural tracts), the differentiation of the HD180 iPSCs was unique. We have previously observed slight differences in differentiation trajectory across other iPSCs carrying mutations associated with different neurodegenerative diseases (data not shown) [6]. These differences highlight a key challenge in comparing the function of iBMEC monolayers: how to optimize differentiation for robust comparisons. Since the yield of adherent BMEC-like cells is dependent on seeding density, we performed differentiations over a wide range of experimental variables to identify conditions where iBMEC adherence and barrier function were optimized. Such analysis of the differentiation protocol is key to reliable assessment of differences in barrier phenotype due to genetic mutations. Given that cerebrovascular microvessels are also comprised of supporting cell types (glia and mural cells), future studies incorporating these iPSC-derived cell types will be needed to unravel cell-type specific contributions to BBB dysfunction.

### Barrier function

While TEER values for HD180 iBMEC monolayers were lower than HD-corrected iBMECs, values for both cell types remained above 500 Ω cm^2^ over 10 days. As previously reported [50], TEER values for iBMECs are not stable on Transwells; HD180 iBMECs displayed increasing TEER over 10 days which could suggest a delayed maturation processes. However, 10 days of culture was not associated with any enrichment of claudin-5 and instead was associated with loss of VE-cadherin junctional immunofluorescence suggesting lost endothelial identity. Consistent with this observation, there were no statistically significant differences in permeability for Lucifer yellow or 10 kDa dextran in 2D or 3D models. Although staining of occludin and ZO-1 at cell–cell junctions was reduced in HD180 iBMECs, these differences had no apparent effect on paracellular barrier function. Claudin-5 immunofluorescence remained robust across both HD-corrected and HD180 iBMECs (as previously observed across other source iPSCs [5, 15, 17]), despite low transcript abundances.

### Beyond barrier function

To explore BMEC phenotypes beyond permeability, we exposed iBMEC monolayers to hydrogen peroxide, VEGF, and mannitol. Our results suggest unique responses of HD180 iBMECs to oxidative, angiogenic, and osmotic stress, which may predispose the BBB to damage during HD progression and highlights potential therapeutic targets. We observed that HD180 iBMECs were more susceptible to hydrogen peroxide-induced injury; antioxidants have been explored for treatment of HD [51], whose effects could be at least partially mediated by BBB protection. Also, we observed that HD180 iBMECs displayed increased VEGFR2 protein expression and were responsive to VEGF exposure by increased sprout density, while limited responsiveness was observed for HD-corrected iBMECs. Further studies are needed to determine whether angiogenic dysfunction is mediated directly by BMECs or through non-cell autonomous effects (i.e. astrocytic release of VEGF) during HD and using in vitro models that better recapitulate angiogenic sprouting activity which is not robust using iBMECs. Lastly, our studies using the hyperosmotic agent mannitol suggest that dynamics of BBB opening could be unique during HD. Given that the relationship between TEER and small molecule permeability is roughly linear and inversely correlated at low TEER values [16, 50, 52], our results suggest that HD180 iBMECs have higher paracellular permeability following osmotic exposure.

### Comparison to iBMECs differentiated from adult HD iPSCs and to BMECs from adult postmortem HD tissue

We confirmed previous reports of GLUT1^+^ and CD31^+^ cells emerging from differentiation of iPSCs harboring expanded CAG repeats. However, our results are in contrast to previous reports using predominately adult HD iBMECs [5]. Previous work utilized a panel of adult iPSCs with CAG lengths of 28, 33, 60, 66, 71, and 109 (juvenile case), which produced TEER values of ~ 4250, ~ 4750, ~ 3500, ~ 2750, ~ 100, ~ 200 Ω cm^2^, respectively. Thus, above 70 CAG repeats TEER values were very low. In contrast, we found average TEER values above ~ 1000 Ω cm^2^ for juvenile HD iBMECs. Our work suggests that changes in barrier function are more nuanced, with 180 CAG repeats still producing cells with high TEER compared to immortalized and primary BMEC cell sources [53]. Findings associated with Wnt signaling are also distinct; studies of predominately adult HD iBMECs indicated aberrantly high Wnt signaling [13, 14], while here we observed depletion of Wnt signaling-related genes in HD180 cells. There are two possible reasons for the observed differences: (1) adult HD iPSCs harbor age-induced epigenetic changes, which could result in unique modes of BBB dysfunction [5], (2) further optimization of the protocol for differentiation of adult HD iBMECs could result in different barrier phenotypes or gene expression. As described above, the differentiation of juvenile HD-iBMECs was optimized for production of neural tracts and endothelial adherence, resulting in relatively high TEER values.

Additionally, recent work characterizing differences in gene expression of cerebrovascular cell types using single nuclei RNA-sequencing of post-mortem tissue [34], did not identify upregulation of Wnt signaling transcripts in HD patients. However, additional studies are needed to understand the time course and CAG length dependence of BBB gene expression changes during HD progression. We observed elevated adhesion of immune cells in HD180 iBMEC microvessels and increased abundance of innate immune activation transcripts (*IL4R*, *CCR1*, *CXCR4*, *IL17RD*, *CXCL12*) despite the lack of external inflammatory stimuli, whereas key initiators and mediators of innate immune activation were upregulated in brain endothelial cells from HD postmortem tissue [34]. Additional studies are needed to identify the mechanisms of innate immune activation in HD BMECs; whereas recent work extending iBMEC differentiation protocols may assist in facilitating these studies in vitro [54].

### Conclusions

In summary, we showed the impact of expanded CAG repeats on iBMEC phenotype using isogenic juvenile HD iPSCs. CAG expansion in juvenile HD180 iBMECs resulted in lower transendothelial electrical resistance, reduced expression of tight junction proteins, and unique gene expression profiles, but no significant changes in paracellular permeability. However, juvenile HD180 iBMECs displayed unique responses to pathological and therapeutic perturbations including angiogenic factors, oxidative stress, and osmotic stress. We also demonstrated that tissue-engineered in vitro BBB models support mechanistic and therapeutic studies of neurodegenerative diseases by exploring unique dynamics of cell turnover and immune cell adhesion. Our results suggest that distinct cerebrovascular changes may occur during juvenile HD that are dependent on the degree of CAG expansion, which should be further explored using isogenic panels that encompass a wider range of CAG repeat lengths [55].

## Supplementary Information

**Additional file 1.** Summary of RNA sequencing data.

**Additional file 2.** Supplemental Information.

## Data Availability

The raw/processed data required to reproduce these findings are available from the corresponding author on reasonable request.
