# Supplementary material for: Brain microvascular endothelial cell dysfunction in an isogenic juvenile iPSC model of Huntington’s disease
Source: Fluids Barriers CNS. 2022 Jun 30;19:54. doi: 10.1186/s12987-022-00347-7 (PMC9245306; doi:10.1186/s12987-022-00347-7)
Supplement: Supplementary file 2 — Additional file 2. Supplemental Information. [file 12987_2022_347_MOESM2_ESM.pdf]

SUPPLEMENTAL INFORMATION

Brain microvascular endothelial cell dysfunction in an isogenic juvenile iPSC model of Huntington's disease

Raleigh M. Linville,[1,2] Renée F. Nerenberg,[1,2] Gabrielle Grifno,[1,2] Diego Arevalo,[1,2] Zhaobin Guo,[1] Peter C. Searson[1,3*]

Supplemental Table 1. iPSC sources.

Supplemental Table 2. Antibodies used in this study.

Supplemental Figure 1. Transcript abundances between HD-corrected and HD180 iPSCs and iBMECs.

Supplemental Figure 2. Additional immunocytochemistry of HD-corrected and HD180 iBMECs.

Supplemental Figure 3. Dysfunction of differentiation trajectory (i.e. cell density and adherent fraction) are independent of initial seeding density, transwell seeding density, and subculture method.

Supplemental Figure 4. Dysfunction of HD180 iBMEC barrier is independent of initial seeding density, transwell seeding density, and subculture method.

Supplemental Figure 5. Dysfunction of HD180 iBMEC barrier is independent of media volume and serum-free alternative medium.

Supplemental Figure 6. Comparison of iBMEC adherent fraction and TEER for all iPSC sources (in ascending CAG repeat length): HD-corrected (HD18), non-isogenic HD21 (Allen Cell Collection), non-isogenic HD50 (NINDS), and HD180. (A-B) Adherent fraction and average TEER values across all cell lines. Data in collected across n = 3 – 10 independent differentiations per cell line.

Supplemental Table 1. iPSC sources.

Cell line	Mutation	Gender	Age isolated	Reprogramming method	Source
CAG180	HTT CAG:180	M	6	lentiviral vector (OCT4, SOX2, KLF4, MYC, NANOG, LIN28)	[7]
HD-corrected	CRISPR-Cas9 corrected clone				
HD50	HTT CAG:50	F	37	Episomal (OCT4, SOX2, KLF4, L-MYC, LIN28)	NINDS cell repository (NN0003930)
HD21	N/A, HTT CAG:21	M	30	Episomal (OCT3/4, SHP53, SOX2, KLF4, L-MYC, LIN28)	Allen Cell Collection (AICS-0023)

Supplemental Table 2. Antibodies used in this study.

Antibody	Vendor	Species	Cat. No	Dilution
CD31	ThermoFisher	Rabbit	RB-10333	1:25
GLUT1	Abcam	Rabbit	115730	1:200
Claudin-5*	Invitrogen	Mouse	35-2500	1:200
Occludin	Invitrogen	Rabbit	40-4700	1:100
ZO1	Invitrogen	Rabbit	402200	1:200
VE-cadherin	R&D Systems	Goat	AF938	1:25
P-gp	Sigma	Mouse	P7965	1:100
VEGFR2	R&D	Mouse	MAB3572	1:100
VCAM1	R&D	Rabbit	AB134047	1:50
ICAM1	Abcam	Mouse	AB2213	1:100
mEM48	Sigma	Mouse	MAB5374	1:100

* Note: immunogen sequence used to generate claudin-5 antibody displays 100% sequence identity with claudin-5, while only 50% sequence identity with claudin-3, and no sequence identity with other claudins.

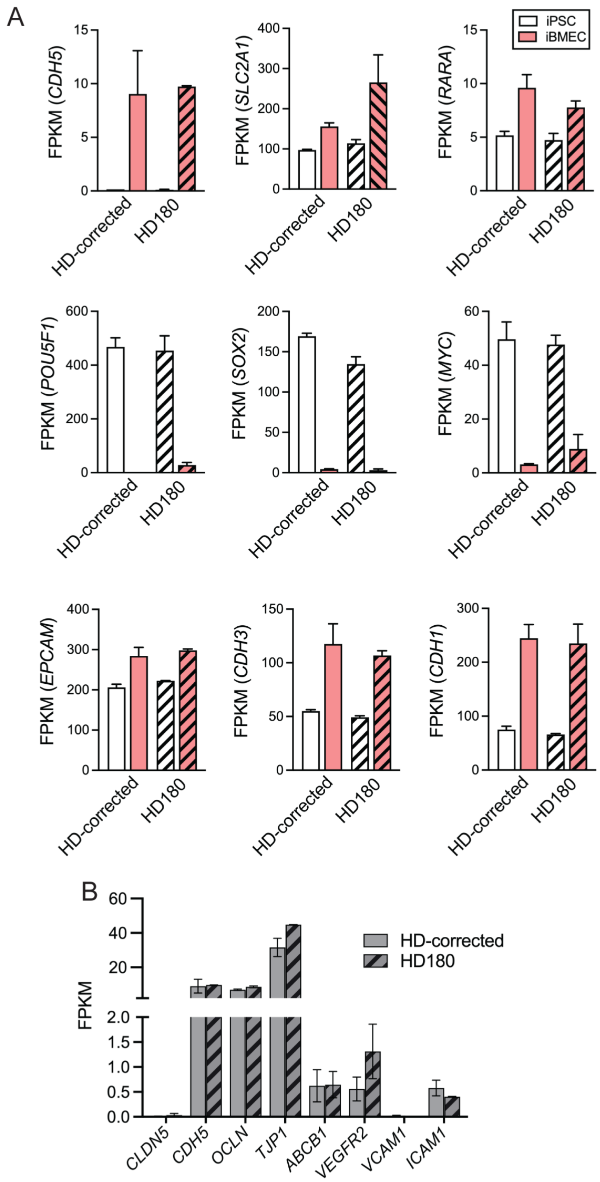

Supplemental Figure 1. Transcript abundances between HD-corrected and HD180 iPSCs and iBMECs. (A) FPKM values for various endothelial, pluripotency, and epithelial genes. (B) Comparison of transcript abundance for BBB markers studied using immunocytochemistry between HD-corrected and HD180 iBMECs; abundances were generally similar. *CLDN5* expression was detectable using qPCR (TaqMan® Assay, #Hs00533949) as previously reported [15, 46], indicating low but not absent mRNA expression (data not shown).

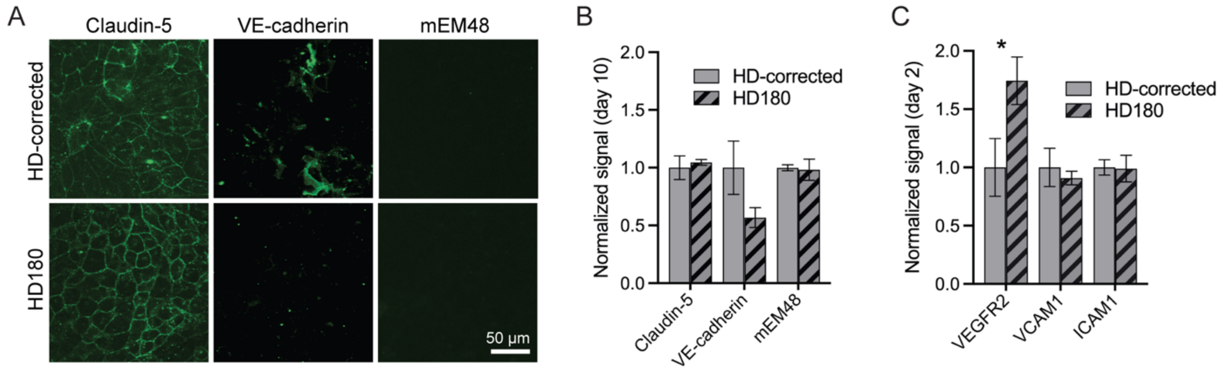

Supplemental Figure 2. Additional immunocytochemistry of HD-corrected and HD180 iBMECs.
(A) Representative immunocytochemistry images of BBB markers (claudin-5, VE-cadherin) and a marker of mutant huntingtin aggregates (mEM48) at day 10.
(B) Semi-quantitative analysis of iBMEC protein expression at day 10 following differentiation. Fluorescence signal was normalized to nuclear signal and then plotted relative to HD-corrected. Data collected across $n = 4$ independent differentiations.
(C) Semi-quantitative analysis of iBMEC protein expression at day 2 following differentiation. Fluorescence signal was normalized to nuclear signal and then plotted relative to HD-corrected. Data collected across $n = 4 – 6$ independent differentiations.

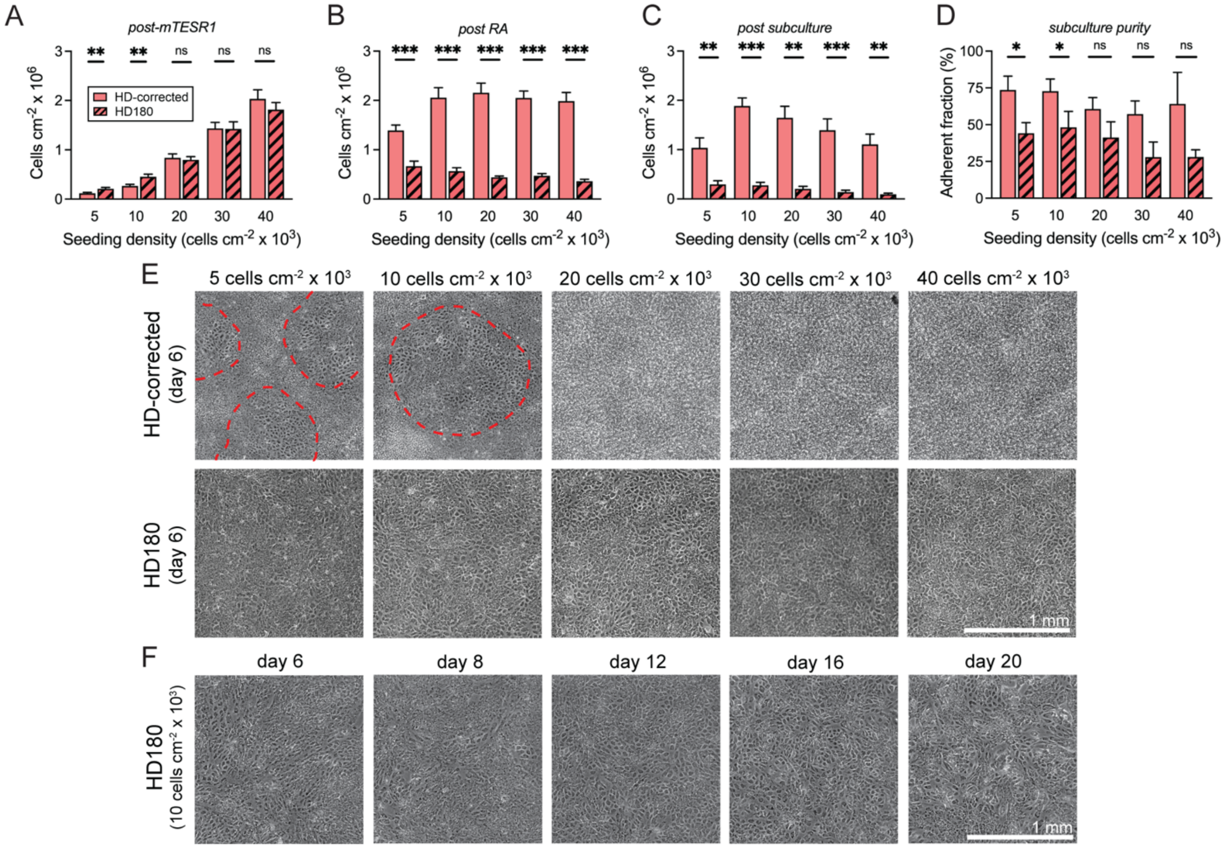

Supplemental Figure 3. Dysfunction of differentiation trajectory (i.e. cell density and adherent fraction) are independent of initial seeding density, transwell seeding density, and subculture method.

(A-C) Cell density over the course differentiation as a function of initial seeding density onto Matrigel-coated plates: (A) at the end of the mTESR1 phase, (B) at the end of the RA phase, and (C) post-subculture. (D) Adherent fraction of iBMECs following subculture. Data in A-C collected across n = 10 (HD-corrected) and 12 (HD180) independent. Data in D collected across *n* = 4 independent differentiations.

(E-F) Dynamics of neural tracts and cell density during iBMEC differentiation. (E) Neural tracts do not form during HD-corrected iBMEC differentiation at non-optimal starting densities (only appear at 5 – 10 cells cm^{-2} x 10^{3} seeding density), while neural tracts do not form during the differentiation of HD180 iPSCs across all starting densities. (F) Neural tracts do not form under extended UM/F- conditions during the differentiation of HD180 iPSCs. Representative images are shown across *n* = 3 independent differentiations.

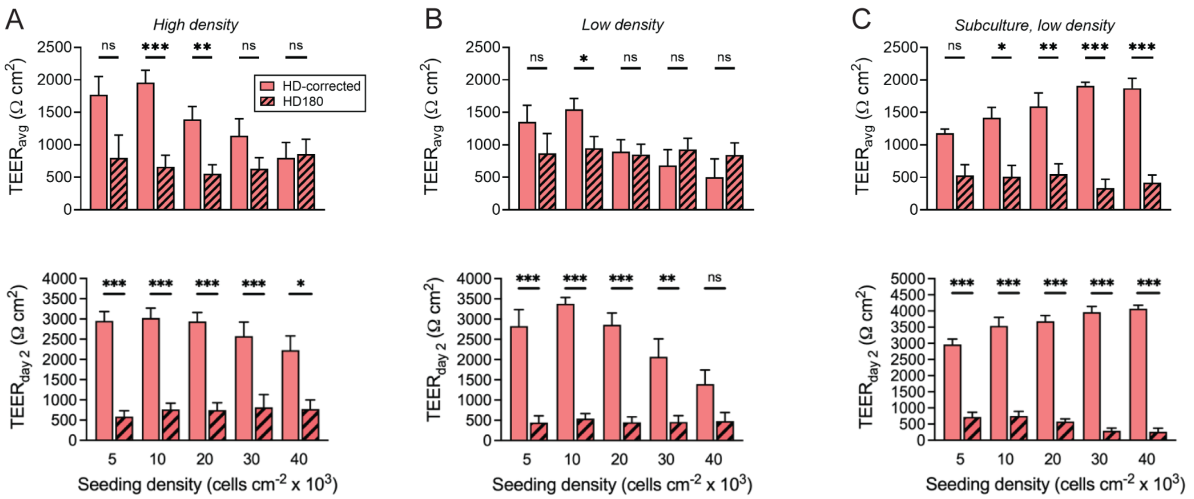

Supplemental Figure 4. Dysfunction of HD180 iBMEC barrier is independent of initial seeding density, transwell seeding density, and subculture method. (A-C) Average over ten days and day 2 transendothelial electrical resistance (TEER) of iBMEC monolayers after seeding at high density (1×10^6 cells cm^{-2}), low density (0.33×10^6 cells cm^{-2}), or post-subculture (0.33×10^6 cells cm^{-2}). Data collected across $n = 3 - 12$ independent differentiations.

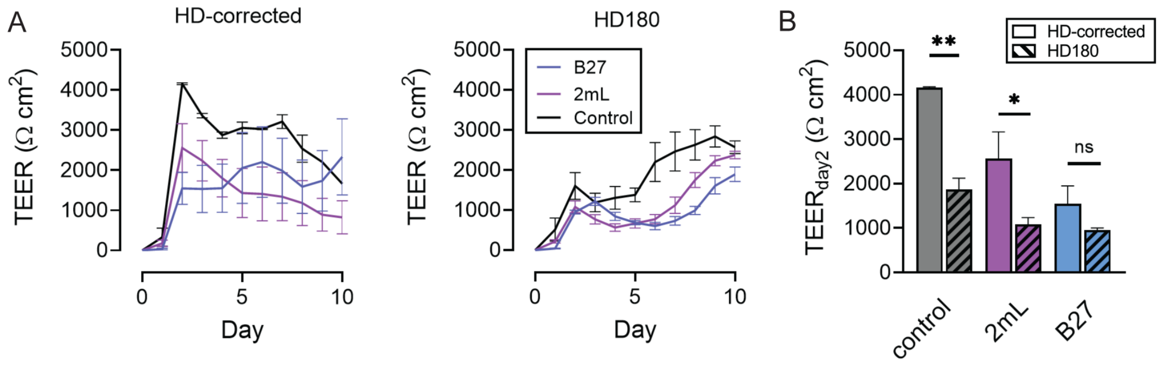

Supplemental Figure 5. Dysfunction of HD180 iBMEC barrier is independent of media volume and serum-free alternative medium.

(A) TEER time course of iBMECs differentiated under control settings, increased media volume (2 mL), or use of B27 as a serum-free alternative.

(B) Day 2 transendothelial electrical resistance (TEER) of iBMEC monolayers across conditions. Data collected across n = 5 (HD-corrected) and 4 (HD180) independent differentiations.

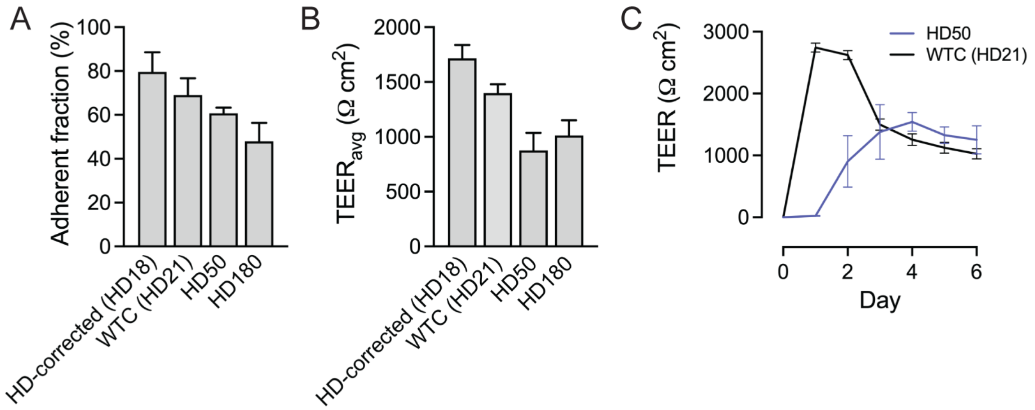

Supplemental Figure 6. Comparison of iBMEC adherent fraction and TEER for all iPSC sources (in ascending CAG repeat length): HD-corrected (HD18), non-isogenic HD21 (Allen Cell Collection), non-isogenic HD50 (NINDS), and HD180. (A-C) Fraction of adherent cells following differentiation, average TEER values, and time course of TEER for non-isogenic iBMECs. Data in collected across *n* = 3 – 10 independent differentiations.
